# Influence of abdominal fat distribution and inflammatory status on post-operative prognosis in non-small cell lung cancer patients: a retrospective cohort study

**DOI:** 10.1007/s00432-024-05633-5

**Published:** 2024-03-03

**Authors:** Mengtian Ma, Muqing Luo, Qianyun Liu, Dong Zhong, Yinqi Liu, Kun Zhang

**Affiliations:** 1 Department of Radiology, The First Hospital of Hunan University of Chinese Medicine, Changsha, 410007 Hunan Province People’s Republic of China; 2 Department of Radiology, The Third Xiangya Hospital, Central South University, Changsha, 410013 Hunan Province People’s Republic of China; 3 Department of Medical Imaging, Yueyang Central Hospital, Yueyang, 414000 Hunan Province People’s Republic of China; 4 Department of Nuclear Medicine, XiangYa Hospital CentralSouth University, Changsha, 410005 Hunan Province People’s Republic of China; 5 College of Integrated Traditional Chinese and Western Medicine, Hunan University of Chinese Medicine, Changsha, 410208 People’s Republic of China

**Keywords:** Non-small cell lung cancer, Visceral fat, Subcutaneous fat, Systemic inflammation

## Abstract

**Purpose:**

To evaluate the influence of visceral fat area (VFA), subcutaneous fat area (SFA), the systemic immune-inflammation index (SII) and total inflammation-based systemic index (AISI) on the postoperative prognosis of non-small cell lung cancers (NSCLC) patients.

**Methods:**

266 NSCLC patients received surgery from two academic medical centers were included. To assess the effect of abdominal fat measured by computed tomography (CT) imaging and inflammatory indicators on patients’ overall survival (OS) and progression-free survival (PFS), Kaplan–Meier survival analysis and Cox proportional hazards models were used.

**Results:**

Kaplan–Meier analysis showed the OS and PFS of patients in high-VFA group was better than low-VFA group (*p* < 0.05). AISI and SII were shown to be risk factors for OS and PFS (*p* < 0.05) after additional adjustment for BMI (Cox regression model II). After further adjustment for VFA (Cox regression model III), low-SFA group had longer OS (*p* < 0.05). Among the four subgroups based on VFA (high/low) and SFA (high/low) (*p* < 0.05), the high-VFA & low-SFA group had the longest median OS (108 months; 95% CI 74–117 months) and PFS (85 months; 95% CI 65–117 months), as well as the lowest SII and AISI (*p* < 0.05). Low-SFA was a protective factor for OS with different VFA stratification (*p* < 0.05).

**Conclusion:**

VFA, SFA, SII and AISI may be employed as significant prognostic markers of postoperative survival in NSCLC patients. Moreover, excessive SFA levels may encourage systemic inflammation decreasing the protective impact of VFA, which may help to provide targeted nutritional support and interventions for postoperative NSCLC patients with poor prognosis.

## Introduction

Lung cancer is the most common cause of cancer-related deaths worldwide, with 2 million new cases and 1.76 million recorded deaths per year (Thai et al. 2021). The majority of lung cancers, approximately 80% to 85% of all lung cancers, are non-small cell lung cancers (NSCLC) (Kirshenboim et al. 2023). At present, surgical intervention remains the primary approach for managing non-small cell lung cancer (Siegel et al. 2023). However, over the extended follow-up periods, it has become evident that NSCLC patients who undergo surgery may still encounter frequent instances of recurrence and metastasis, ultimately impacting their overall survival (Alduais et al. 2023). Therefore, it is urgent to search for effective prognostic indicators, which is conducive to providing basis for diagnosis and treatment of NSCLC and improving the survival rate of patients.

The prognosis of lung cancer patients is influenced not only by cancer-related factors, such as disease stage, pathological type, and tumor cell differentiation, but also by patient-specific factors like muscle mass, obesity, and lifestyle habits (Friedenreich et al. 2021; Thai et al. 2021). While there is extensive research on sarcopenia as a factor in the patient-specific domain, existing studies indicate that lung cancer patients with concurrent sarcopenia experience more adverse reactions during treatment and have a poorer prognosis (Voorn et al. 2022). In recent decades, changes in human lifestyle have contributed to increasing concern over obesity, which has been found to be a substantial risk factor for various cancers (Kolb et al. 2016; Avgerinos et al. 2019; Shao et al. 2022).. However, some studies indicate that obese lung cancer patients may exhibit improved responses to chemotherapy and immunotherapy (Cortellini et al. 2020; Collet et al. 2021). Conventional assessments of obesity primarily rely on BMI; however, individuals with the same BMI may possess different amounts of body fat (Silveira et al. 2021). Compared to BMI, measurements of visceral fat and subcutaneous fat provide a more accurate representation of fat distribution in patients (Tabuso et al. 2017). Abdominal computed tomography (CT) scans are commonly employed for quantifying visceral fat, and as lung cancer patients frequently undergo CT scans, it is feasible to evaluate body composition based on CT imaging (van Vugt et al. 2017). Research has indicated that visceral and subcutaneous fat have distinct functions and play diverse roles in cancer occurrence and progression, sometimes even exerting opposing effects (Lee et al. 2018; Buckley et al. 2022). Similarly, the prognostic value of visceral and subcutaneous fat for postoperative survival in lung cancer patients remains a subject of debate (Lee et al. 2022a; Gezer et al. 2023), and the interplay among obesity markers in the prognosis of NSCLC remains unclear.

A strong association between obesity and tumor development has been established, characterized by chronic inflammation and alterations in immune cell populations (Rathmell 2021). Moreover, existing studies have confirmed that some systemic inflammatory indicators can comprehensively reflect the inflammation and immune status of patients with various malignant tumors (Lalani et al. 2018; Russo et al. 2020; He et al. 2022). For instance, the immunological status can be reflected by the Glasgow Prognostic Score (GPS) and C-reactive protein (CRP) (Laird et al. 2013; Nost et al. 2021). However, the potential of the systemic immune-inflammation index (SII) and total inflammation-based systemic index (AISI) as novel composite measurements of peripheral blood cells for evaluating postoperative survival prognosis in NSCLC patients, as well as their interplay with visceral and subcutaneous fat, remains poorly understood.

In this study, we examined the predictive value of visceral fat, subcutaneous fat, and systemic inflammatory markers in postoperative NSCLC patients. Furthermore, we sought to explore the relationships between visceral fat, subcutaneous fat, and systemic inflammation in NSCLC patients in order to shed light on the underlying mechanisms involved.

## Materials and methods

Between January 2012 and December 2020, 266 postoperative patients with stage I to stage II NSCLC were included in this retrospective cohort research at Yueyang Central Hospital and the First Hospital of Hunan University of Chinese Medicine. The ethics committees of the two academic medical centers both authorized the retrospective cohort research. Given the nature of the study and the utilization of data obtained from routine clinical practice, the requirement for informed consent and signature was waived.

Inclusion criteria consisted of: (1) histopathological confirmation of stage I–II NSCLC and treatment with radical surgery alone; (2) absence of acute infection or fever within the two weeks prior to admission; (3) no history of chronic infectious diseases, immune-related disorders, or other malignancies; and (4) complete clinical, imaging, and pathological data. Exclusion criteria consisted of: (1) histopathological diagnosis of concurrent small cell lung cancer; (2) abnormal levels of C-reactive protein, albumin, or complete blood count due to other diseases; or (3) received neoadjuvant radiotherapy or chemotherapy. Clinical management and laboratory assessments followed the NCCN Clinical Practice Guidelines in Oncology (NCCN Guidelines, Version 3.2022) for NSCLC (Ettinger et al. 2023).

### CT examination

Non-enhanced abdominal CT scans were conducted within one month prior to surgery using a 64-slice multidetector CT scanner (Philips Brilliance; Philips) or 128-slice multidetector CT scanner (Siemens Somatom Definition; Siemens). Prior to the examination, patients were advised to fast for 6–8 h and were positioned in the supine position. The scanning range encompassed both kidneys and the L3 vertebral body, with specific scanning parameters outlined in Table 1.

**Table 1:** Technical Parameters of CT Examination

Parameter	Institution 1 Philips Brilliance	Institution 2 Siemens SOMATOM Definition
Tube voltage (kVp)	120	120
Tube current (mA)	240	225
Rotation time (s)	0.5	0.5
Beam collimation (mm)	64 × 0.625	64 × 0.6
Acquisition mode	Helical	Helical
Section thickness (mm)	5	5
Section interval (mm)	5	5
Kernel	B	B31f
Reconstruction algorithm	iDose4 level 4	SAFIRE S3
Matrix	512 × 512	512 × 512
Field of view (mm × mm)	350 × 350	330 × 330

Institution 1 indicates The First Hospital of Hunan University of Chinese Medicine, institution 2 indicates Yueyang Central Hospital

### Image analysis

**Fig. 1:**
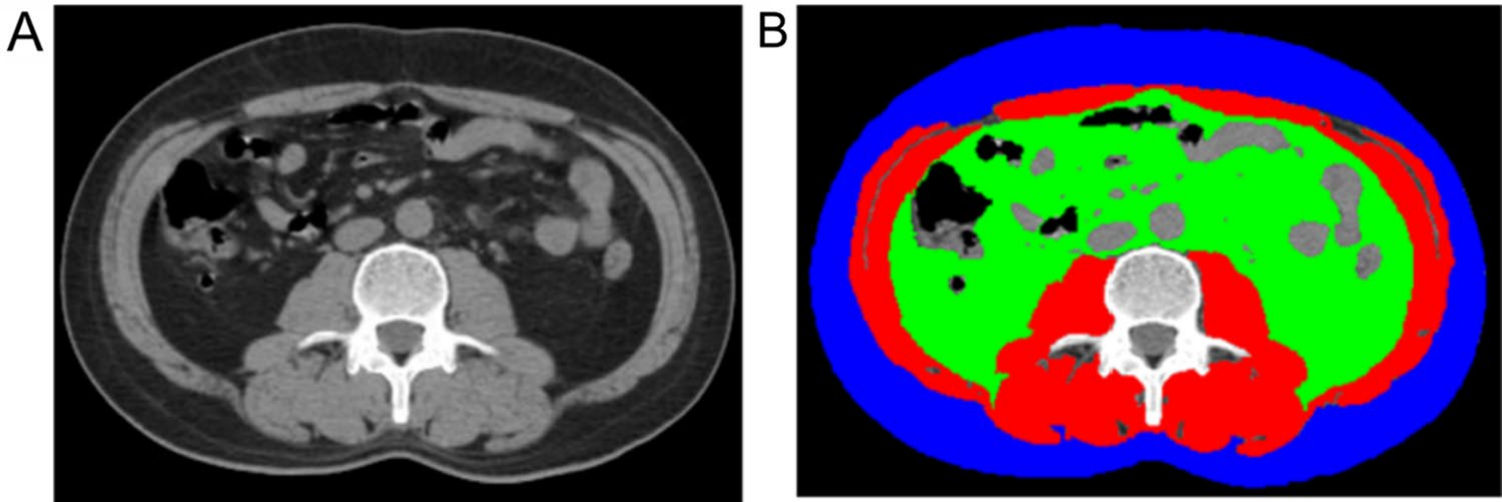
Example of threshold-based delineation of fat and skeletal muscle parameters on abdominal CT images at the 3rd lumbar vertebral level (blue, subcutaneous fat area; green, abdominal visceral fat area; red, abdominal wall and lumbar muscle area)

Image data were transferred to a local PACS workstation, where two radiologists, blind to clinical data and outcomes, selected the axial non-contrast CT image at the mid-level of the L3 vertebral body as the anatomical map for region of interest (ROI) determination. The selected image exhibited no artifacts between muscle and surrounding tissues, as well as visceral fat, intestines, or other organs. ImageJ 1.51j8 software (https://imagej.nih.gov/ij/) (Schneider et al. 2012; Gomez-Perez et al. 2016) was employed to measure subcutaneous fat area (SFA), visceral fat area (VFA), and skeletal muscle area (SMA) on the selected CT image (Fig. 1). A threshold range of − 190 to − 30 Hounsfield units (HU) was used to identify fat tissue (Nitsche et al. 2022), while a range of − 29 to + 150 HU was used to identify muscle tissue, including the psoas, erector spinae, quadratus lumborum, transversus abdominis, internal and external oblique muscles, and rectus abdominis (Gomez-Perez et al. 2016). Manual contour correction was performed independently by a trained radiologist, and intra-organ fat (such as renal intra-organ fat) was excluded.

The skeletal muscle index (SMI) was calculated as the SMA divided by height squared. CT-determined sarcopenia was defined as an SMI of ≤ 52.4 cm^2^/m^2^ in men and ≤ 38.5 cm^2^/m^2^ in women, as proposed by a CT-based sarcopenia study of patients with cancer (Prado et al. 2008; Ashton et al. 2023). The measurements and calculations of SFA, VFA, SMA, and SMI parameters were independently performed by the two aforementioned physicians.

### Clinical variables and endpoints

According to the Asia–Pacific guidelines, body mass index (BMI) was computed as weight divided by the square of height (kg/m^2^) and classified as underweight (< 18.5 kg/m^2^), normal weight (18.5–22.9 kg/m^2^), overweight (23–24.9 kg/m^2^), or obese (≥ 25 kg/m^2^) (Lee et al. 2022b). Patients were categorized into high and low VFA groups, as well as high and low SFA groups, using the median values of VFA and SFA as cutoff points. SII was calculated as the product of platelet count and neutrophil count divided by the lymphocyte count, while AISI was calculated as the product of neutrophil count, platelet count, and monocyte count divided by lymphocyte count. All patients were regularly followed after discharge, and detailed records were kept regarding treatment outcomes, tumor recurrence, time of recurrence, and patient survival status. The prognostic value of each parameter was assessed in terms of overall survival (OS) and progression-free survival (PFS). OS referred to the time interval between the diagnosis of the illness and death from any cause. The time between a diagnosis and the first sign of progression (local or distant) or death from any cause is known as PFS, whichever occurred earlier, excluding patients with stage III-IV or missing progression data. Failure to follow up with the patient > 3 times by any means on non-simultaneous days after the specified time is recorded as a lost visit. The last date for follow-up was set as January 1, 2023.

### Statistical analysis

R software (version 4.2.0, Boston, MA, USA) was used for the statistical analysis. The normality of continuous variables was assessed using the Shapiro–Wilk test. Non-normally distributed continuous variables were reported as median (interquartile range [IQR]), while normally distributed continuous variables were presented as mean ± standard deviation. Categorical variables were expressed as frequencies. The effects of VFA (high/low), SFA (high/low), BMI (underweight/normal/obese/overweight), and sarcopenia (with/without) on patient OS and PFS were assessed using Kaplan–Meier analysis and the log-rank test. The Cox proportional hazards model was utilized to investigate the influence of BMI, VFA, SFA, AISI (continuous, per 100 × 10 18/L^2^), and SII (continuous, per 100 × 10 9/L) on the prognosis of NSCLC patients, with adjustment for confounders. In Cox regression models I, II, III, IV and V, baseline adjustments were made for age (continuous per year), gender (male/female), TNM stage (I/II), and sarcopenia. Furthermore, in models II, III, IV and V, additional adjustments were made for BMI, VFA, inflammatory index (AISI, SII) and SFA. Variables with a p value < 0.05 in model I were included in models II, III, IV and V to determine whether their prognostic value depended on BMI, VFA, SII and AISI. Kaplan–Meier analysis and the log-rank test were utilized to compare the prognostic differences among different subgroups based on VFA and SFA, while the hierarchically Cox proportional hazards model was used to investigate the impact of SFA on patient prognosis across various VFA strata. The Kruskal–Wallis test was used to compare the differences in AISI and SII among multiple groups. Pairwise comparisons were performed using Dunn’s multiple comparison test. A p value of < 0.05 was considered statistically significant for all analyzes.

## Results

### Patient characteristics

In this study, a total of 266 patients (IQR 55–69 years; 76 females and 190 males) were included, including 5 underweight patients (1.9%), 115 patients with normal BMI (43%), 79 overweight patients (30%), and 67 patients with obesity (25%). CT-diagnosed sarcopenia was present in 76 patients (29%) based on the defined cutoff value. Using the median values of VFA and SFA as cutoffs, the patients were divided into high VFA (≥ 111 cm^2^, n = 133, 50.0%) and low VFA (< 111 cm^2^, n = 133, 50.0%) groups, as well as high SFA (≥ 105cm^2^, n = 134, 50%) and low SFA (< 105 cm^2^, n = 132, 50%) groups. During the postoperative follow-up period, the median OS for all patients was 51 months (IQR 39–72months) and the median PFS was 47 months (IQR 33–67 months) (Table 2).

**Table 2:** Baseline characteristics of patients

Characteristic	Patients (N = 266)
Age (years) *	62 (55, 69)
Gender (%)	
Female	76 (29%)
Male	190 (71%)
TNM stage (%)	
I	128 (48%)
II	138 (52%)
Type of surgery	
Wedge resection	9 (3.4%)
Lobectomy	202 (76%)
Segmental resection	55 (21%)
BMI (kg/m^2^) *	23.12 (21.27, 24.66)
Underweight (< 18.5)	5 (1.9%)
Normal (18.5–22.9)	115 (43%)
Overweight (23.0–24.9)	79 (30%)
Obese (≥ 25)	67 (25%)
SMI (cm^2^/m^2^)*	53 (47, 58)
Sarcopenia (%)	
With	76 (29%)
Without	190 (71%)
VFA (cm^2^)*	111 (76, 160)
High	133 (50%)
Low	133 (50%)
SFA (cm^2^)*	105 (81, 133)
High	134 (50%)
Low	132 (50%)
AISI*	210 (121, 370)
SII*	479 (330, 682)
Pathology	
Adenocarcinoma	165 (62%)
Squamous carcinoma	101 (38%)
PFS (month)	47 (33, 67)
OS (month)	51 (39, 72)

*BMI* body mass index, *SMI* skeletal muscle index, *VFA* visceral fat area, *SFA* subcutaneous fat area, *AISI* Aggregate Index of Systemic Inflammation, *SII* systemic immune-inflammation index, *OS* overall survival, *PFS* progression-free survival

*Median (IQR)

### Kaplan–Meier estimates of OS in NSCLC patients

**Fig. 2:**
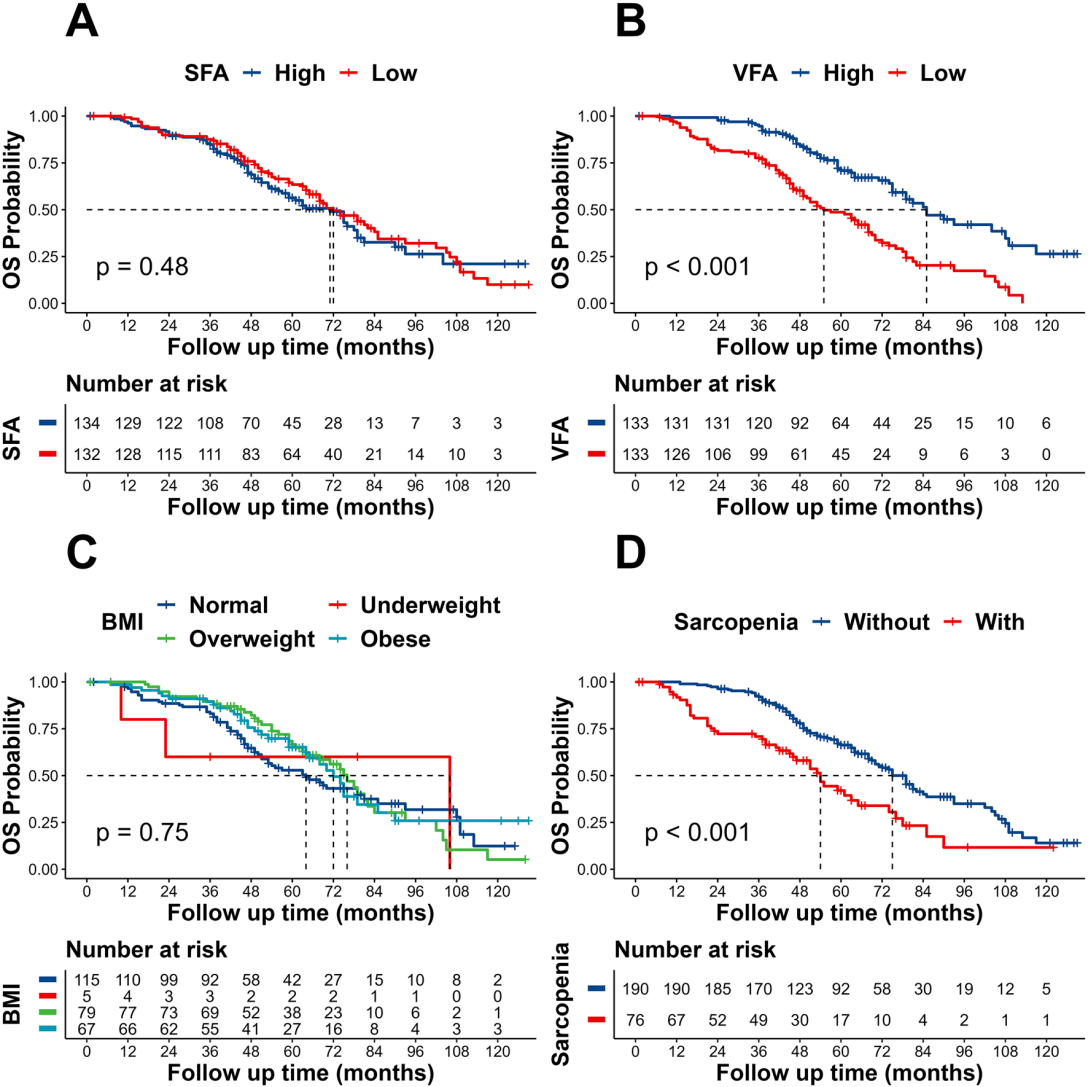
Kaplan–Meier estimates of overall survival, according to SFA (**A**), VFA (**B**), BMI (**C**) and Sarcopenia (**D**). *OS* overall survival, *BMI* body mass index, *VFA* visceral fat area, *SFA* subcutaneous fat area

No significant difference in OS was observed among different BMI subgroups (under-weight, normal weight, overweight, or obese) (log-rank *p* = 0.75). As compared to patients with sarcopenia (median OS 57 months; 95% CI 46–74 months), patients without sarcopenia had a longer median OS (75 months; 95% CI 70–85 months) (log-rank *p* < 0.001). Additionally, patients with high VFA had a median OS that was 85 months, which was longer than patients with low VFA, who had a median OS of 55 months (log-rank *p* < 0.001). However, as shown in Fig. 2, there was no discernible difference in OS between patients with high SFA (median OS 71 months; 95% CI 59–79 months) and low SFA (median OS 72 months; 95% CI 65–85 months) (log-rank *p* = 0.48).

### Kaplan–Meier estimates of PFS in NSCLC patients

**Fig. 3:**
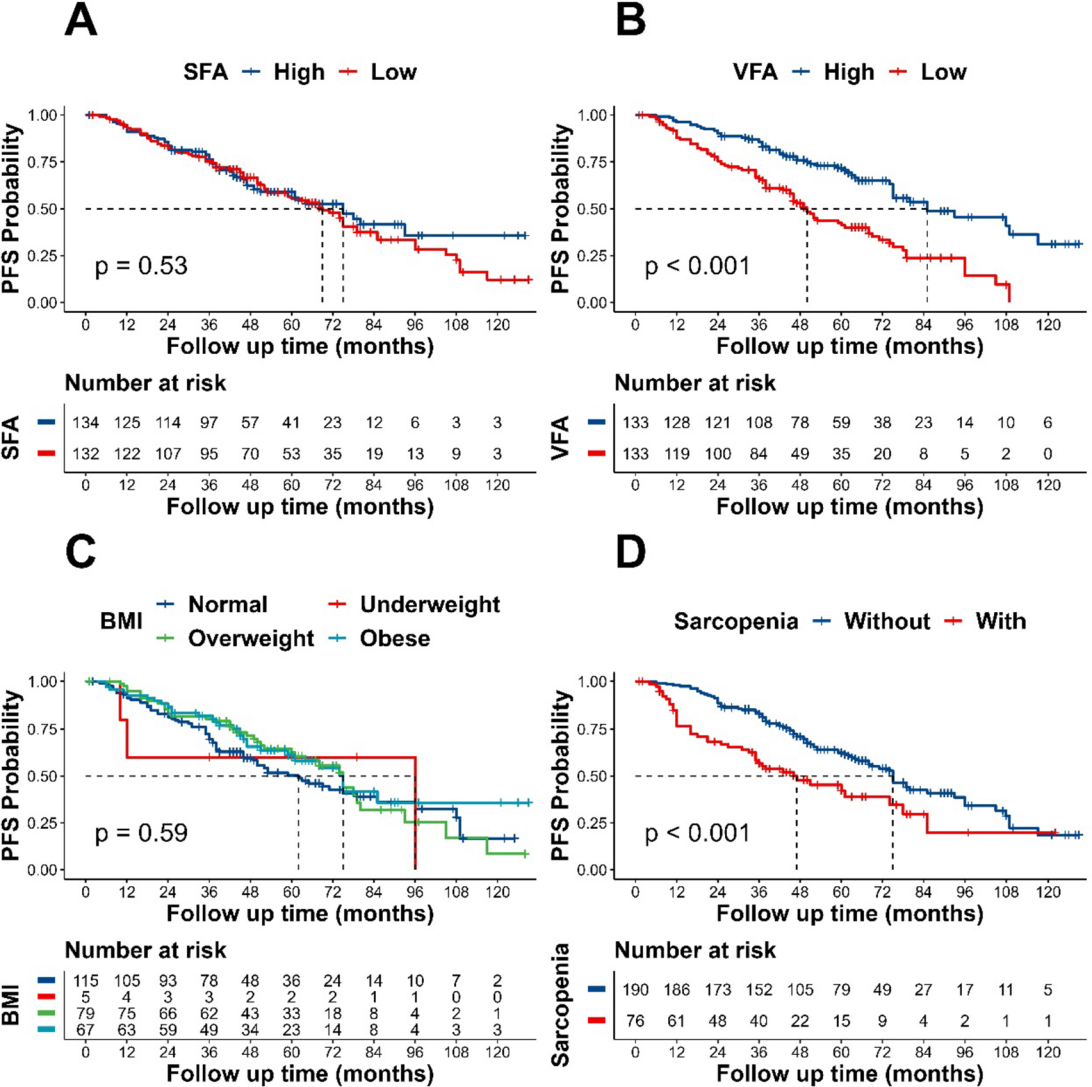
Kaplan–Meier estimates of progression-free survival, according to SFA (**A**), VFA (**B**), BMI (**C**) and Sarcopenia (**D**). *PFS* progression-free survival, *BMI* body mass index, *VFA* visceral fat area, *SFA* subcutaneous fat area

There was no discernible difference in PFS between subgroups based on SFA (high/low) according to Kaplan–Meier curves and log-rank testing (log-rank *p* = 0.53). Patients with high VFA, in contrast, had longer PFS (median PFS 85 months; 95% CI 75–129 months) than patients with low VFA (median PFS 50 months; 95% CI 46–68 months) (log-rank *p* < 0.001). Patients without sarcopenia had longer PFS (mean PFS 75 months; 95% CI 68–93 months) than those with sarcopenia (mean PFS 47 months; 95% CI 35–78 months) (log-rank *p* < 0.001), as shown in Fig. 3.

### Relationship between body composition features, inflammatory markers, and OS, PFS

In the Cox proportional hazards model, BMI, VFA, SFA, AISI, and SII were included, adjusting for age, gender, TMN stage, and sarcopenia (with/without). Cox regression analysis revealed no evidence of a significant relationship between various BMI groups and OS or PFS (*p* > 0.05), even after further adjustment for VFA, AISI, and SII. AISI and SII were shown to be risk factors for OS and PFS (*p* < 0.05) in Cox regression model I, but higher VFA emerged as a protective factor for OS and PFS in NSCLC patients (*p* < 0.05). Further adjustment for BMI (Cox regression model II) did not change the predictive significance of VFA, AISI, and SII. AISI and SII were significant risk factors for OS in NSCLC patients (*p* < 0.05) while AISI and SII remained significant risk factors for PFS (*p* < 0.05) after further adjustment for VFA (Cox regression model III). In Cox regression model IV, VFA remained a protective factor for patient prognosis after additional adjustment for AISI and SII (*p* < 0.001). With additional adjustment for SFA (Cox regression model V), the predictive value of VFA, AISI, and SII remained (*p* < 0.05), as summarized in Tables 3 and 4.

**Table 3:** Association between BMI, VFA, SFA, AISI, SII, and overall survival

Characteristic	Model I		Model II		Model III			Model IV		Model V		
	HR (95% CI)^1^	p value	HR (95% CI)^1^	p value	HR (95% CI)^1^		p value	HR (95% CI)^1^	p value	HR (95% CI)^1^		p value
BMI												
Underweight	1.181 (0.356, 3.915)	0.786			1.051 (0.312, 3.537)		0.936	1.390 (0.419, 4.610)	0.590	1.173 (0.353, 3.897)		0.795
Normal	Ref				Ref			Ref		Ref		
Overweight	0.836 (0.559, 1.252)	0.385			0.979 (0.647, 1.481)		0.920	0.716 (0.470, 1.090)	0.119	0.831 (0.552, 1.252)		0.376
Obese	0.767 (0.495, 1.190)	0.236			1.103 (0.696, 1.749)		0.676	0.723 (0.465, 1.125)	0.150	0.761 (0.486, 1.192)		0.233
VFA												
High	Ref		Ref					Ref		Ref		
Low	2.545 (1.785, 3.628)	< 0.001	2.601 (1.792, 3.776)	< 0.001				2.544 (1.783, 3.631)	< 0.001	3.193 (2.151, 4.739)		< 0.001
SFA												
High	Ref		Ref		Ref			Ref				
Low	1.023 (0.719, 1.455)	0.899	0.969 (0.674, 1.394)	0.867	0.598 (0.403, 0.887)		0.011	1.111 (0.778, 1.587)	0.563			
AISI	1.235 (1.085, 1.406)	0.001	1.274 (1.115, 1.456)	< 0.001	1.216 (1.067, 1.386)		0.003			1.242 (1.090, 1.416)		0.001
SII	1.225 (1.060, 1.417)	0.006	1.249 (1.081, 1.444)	0.003	1.177 (1.014, 1.366)		0.032			1.228 (1.062, 1.420)		0.006

Model II was adjusted for covariates plus BMI (underweight/normal/obese/overweight)

Model III was adjusted for covariates plus VFA (high/low)

Model IV was adjusted for covariates plus AISI (continuous) and SII (continuous)

Model V was adjusted for covariates plus SFA (high/low)

^*1*^*HR* hazard ratio, *CI* confidence interval, *BMI* body mass index, *VFA* visceral fat area, *SFA* subcutaneous fat area, *AISI* Aggregate Index of Systemic Inflammation, *SII* systemic immune- inflammation index

*All models were adjusted for the following covariates: age (continuous), gender (male/female), TMN stage (I/II), sarcopenia (with/ without)

**Table 4:** Association between BMI, VFA, SFA, AISI, SII, and progression-free survival

Characteristic	Model I		Model II		Model III			Model IV			Model V		
	HR (95% CI)^1^	p value	HR (95% CI)^1^	p value	HR (95% CI)^1^		p value	HR (95% CI)^1^		p value	HR (95% CI)^1^		p value
BMI													
Underweight	1.087 (0.328, 3.598)	0.891			0.975 (0.290, 3.273)		0.967	1.242 (0.375, 4.107)		0.723	1.137 (0.341, 3.788)		0.834
Normal	Ref				Ref			Ref			Ref		
Overweight	0.836 (0.556, 1.257)	0.389			0.981 (0.647, 1.488)		0.930	0.726 (0.475, 1.110)		0.140	0.872 (0.575, 1.322)		0.518
Obese	0.728 (0.465, 1.139)	0.164			1.052 (0.654, 1.693)		0.835	0.695 (0.443, 1.090)		0.113	0.768 (0.484, 1.217)		0.261
VFA													
High	Ref		Ref					Ref			Ref		
Low	2.545 (1.785, 3.628)	< 0.001	2.601 (1.792, 3.776)	< 0.001				2.535 (1.777, 3.619)		< 0.001	3.193 (2.151, 4.739)		< 0.001
SFA													
High	Ref		Ref		Ref			Ref					
Low	1.272 (0.890, 1.819)	0.186	1.241 (0.856, 1.801)	0.254	0.811 (0.542, 1.213)		0.307	1.385 (0.965, 1.988)		0.077			
AISI	1.211 (1.064, 1.378)	0.004	1.239 (1.086, 1.414)	0.001	1.190 (1.044, 1.356)		0.009				1.227 (1.078, 1.396)		0.002
SII	1.170 (1.008, 1.357)	0.039	1.187 (1.024, 1.375)	0.023	1.123 (0.963, 1.308)		0.139				1.176 (1.015, 1.362)		0.031

Model II was adjusted for covariates plus BMI (Underweight / Normal / Obese / Overweight)

Model III was adjusted for covariates plus VFA (high/low)

Model IV was adjusted for covariates plus AISI (continuous) and SII (continuous)

Model V was adjusted for covariates plus SFA (High / Low)

^*1*^*HR* hazard ratio, *CI* confidence interval, *BMI* body mass index, *VFA* visceral fat area, *SFA* subcutaneous fat area, *AISI* Aggregate Index of Systemic Inflammation, *SII* systemic immune- inflammation index

*All models were adjusted for the following covariates: age (continuous), gender (male/female), TMN stage (I/II), Sarcopenia (with/without)

### Stratified analysis based on VFA and SFA in NSCLC patients

**Fig. 4:**
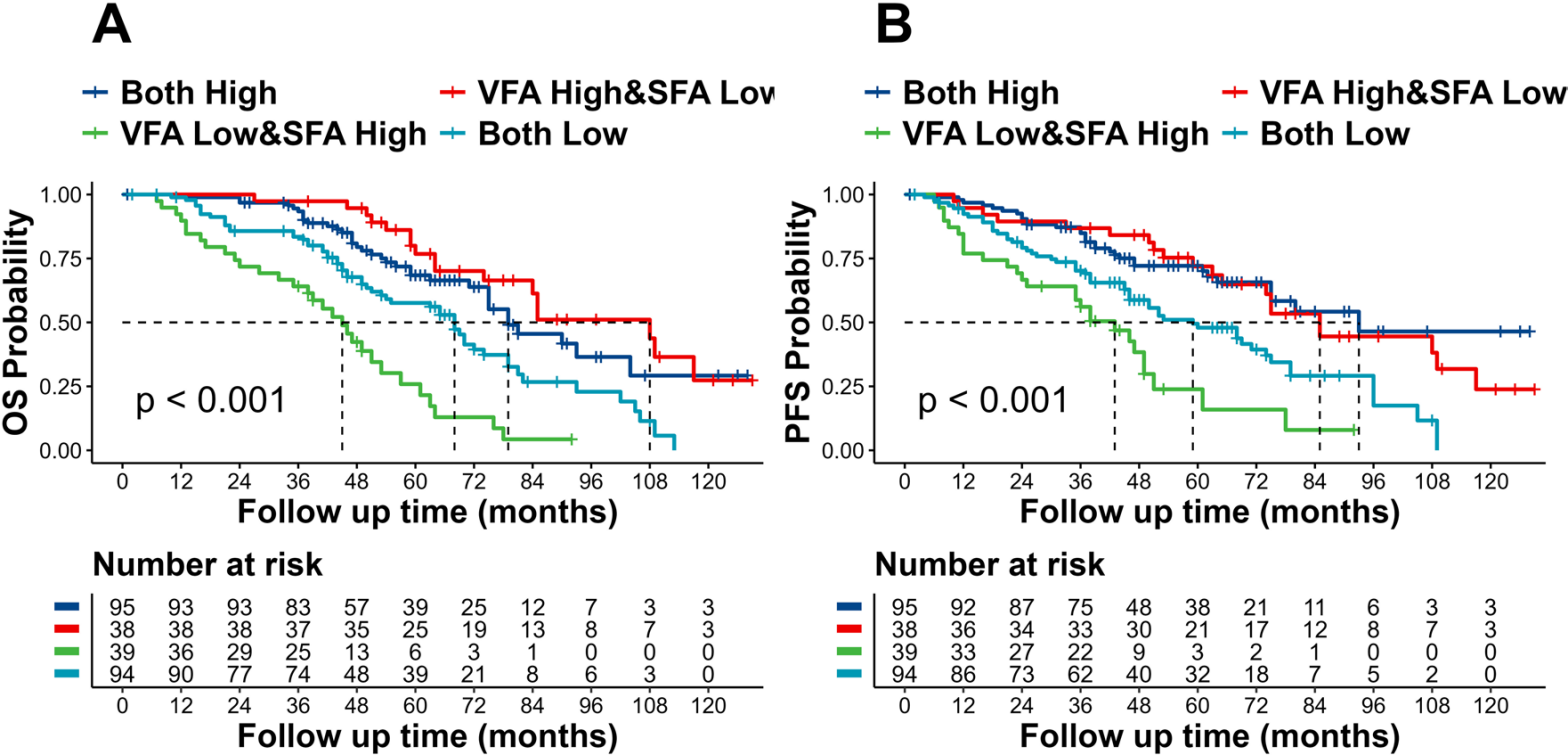
Compared survival difference for OS (**A**) and PFS (**B**) based on VFA (high/low) and SFA (high/low). *OS* overall-survival, *PFS* progression-free-survival, *VFA* visceral fat area, *SF A* subcutaneous fat area

Subsequent analysis involved stratifying the patients into four subgroups based on VFA (high/low) and SFA (high/low): high VFA & low SFA group, low VFA &high SFA group, both high group, and both low group. Figure 4 shows the results of a Kaplan–Meier analysis and log-rank tests, which showed notable differences in OS and PFS among these four subgroups (log-rank *p* < 0.001). Notably, the high VFA & low SFA group exhibited the longest median OS (108 months; 95% CI 74–117 months) and PFS (85 months; 95% CI 65–117 months), accompanied by a 5-year OS rate of 65.79%. Conversely, the low VFA & high SFA group displayed the shortest median OS (45 months; 95% CI 38–57 months) and PFS (43 months; 95% CI 35–51 months), with a 5-year OS rate of 15.38%.

**Fig. 5:**
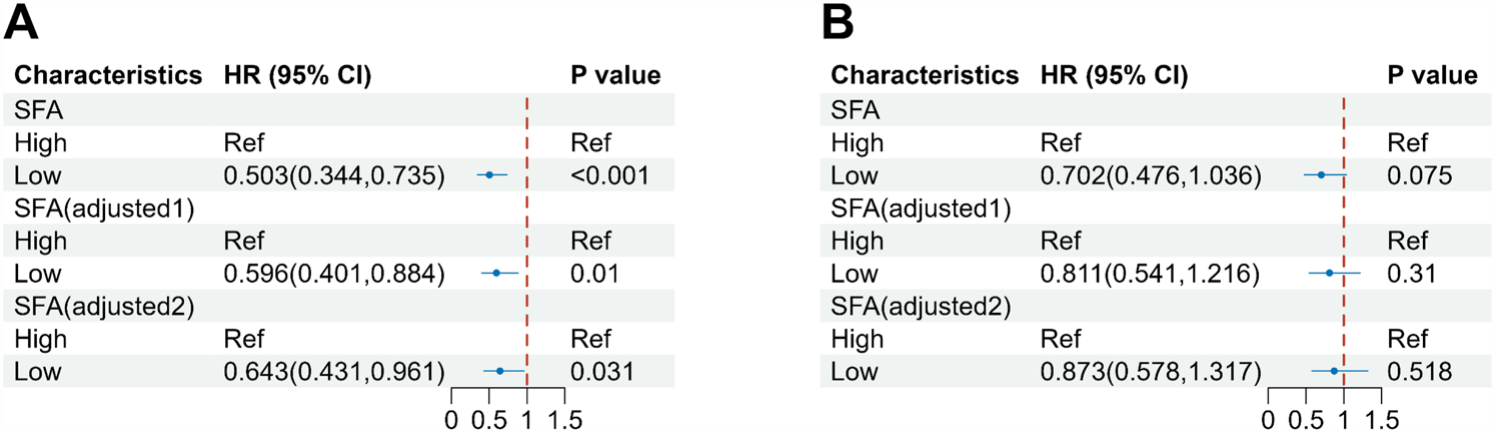
Cox regression analysis of SFA with stratification by VFA. **A** Hierarchically Cox regression for OS; **B** Hierarchically Cox regression for PFS. Adjusted 1 was adjusted for the following covariates: Age (continuous), Gender (Male/Female), TMN stage (I/II), Sarcopenia (With/Without); adjusted 2 was adjusted for covariates plus AISI (continuous) and SII (continuous). *VFA* visceral fat area, *SFA* subcutaneous fat area, *AISI* Aggregate Index of Systemic Inflammation, *SII* systemic immune- inflammation index

To further analyze the effect of SFA on prognosis under different VFA stratification, Cox regression analysis was performed after correcting for different variables. Results showed that low SFA was a protective factor for patients with OS under different VFA (high/low) groups (*p* < 0.05). Adjusted for age, sex, TMN stage, and sarcopenia (adjusted 1), low SFA was still a protective factor for OS (*p* < 0.05). Further adjustment for AISI and SII, the predictive value of SFA remained (*p* < 0.05), as summarized in Fig. 5.

### Differences in inflammatory markers among different groups based on VFA and SFA

**Fig. 6:**
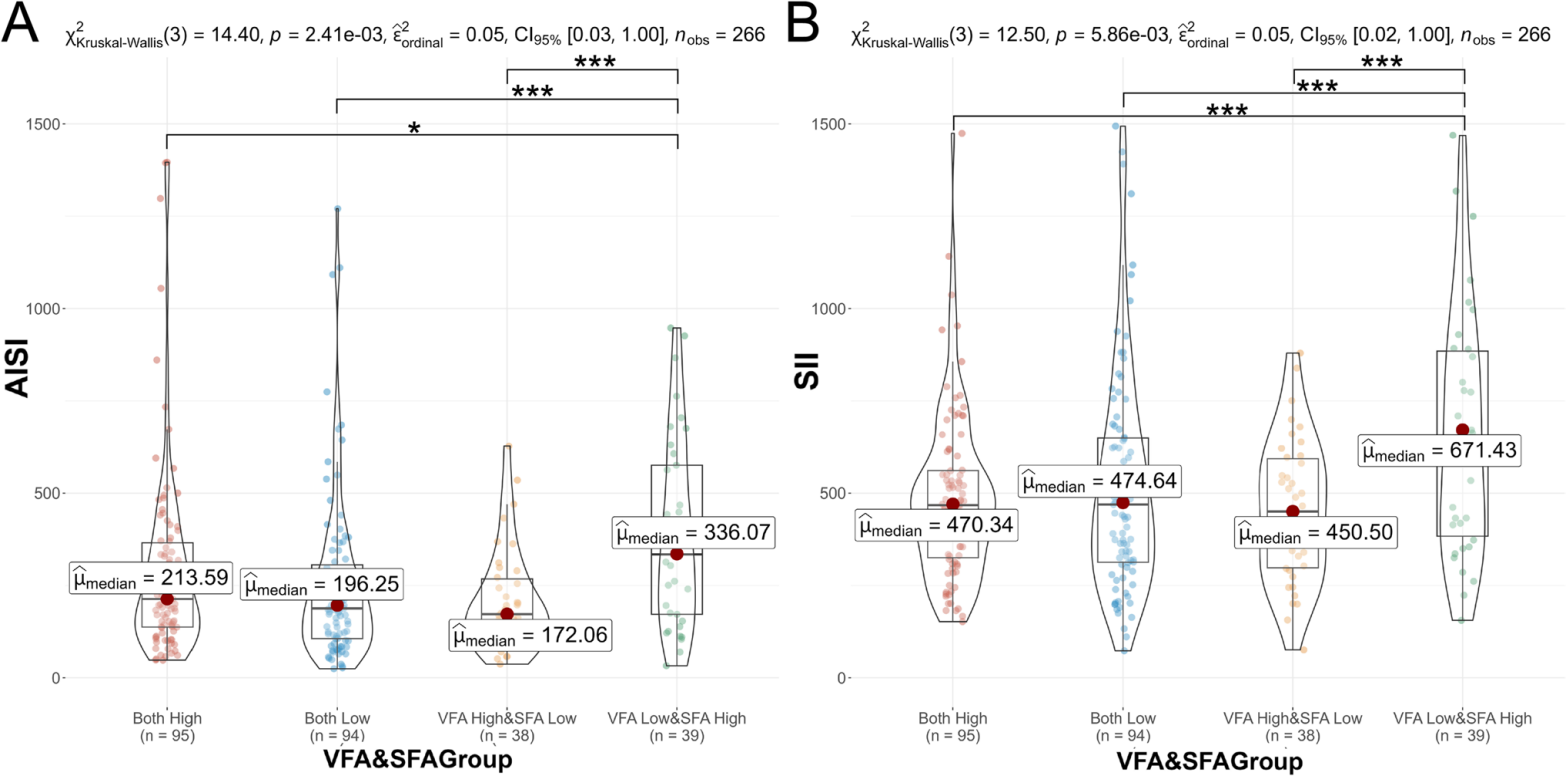
Compared difference for AISI (**A**) and SII (**B**) based on VFA (high/low) and SFA (high/low). *VFA* visceral fat area, *SFA* subcutaneous fat area, *AISI* Aggregate Index of Systemic Inflammation, *SII* systemic immune-inflammation index. *p < 0.05; **p < 0.01; ***p < 0.001

SII and AISI values were significantly different among the above four subgroups based on VFA and SFA levels (*p* < 0.01). The high VFA & low SFA group exhibited the lowest median SII (450.50) and AISI (172.06), while the low VFA & high SFA group displayed the highest median SII (671.43) and AISI (336.07). In pairwise comparisons, differences of AISI and SII values were statistically significant between the both high group and the low VFA & high SFA group, the both low group and the low VFA & high SFA group, as well as the high VFA & low SFA group and the low VFA & high SFA group (all *p* < 0.05), as shown in Fig. 6.

## Discussion

The primary and optimal treatment for early-stage NSCLC is curative surgical resection (Alduais et al. 2023). However, postoperative risks such as recurrence, metastasis, and mortality still exist. We conducted a retrospective cohort study including 266 patients to comprehensively analyze factors affecting the prognosis of NSCLC patients after surgery. Our findings indicated that VFA and SFA, but not BMI, were associated with postoperative prognosis, and that SII and AISI, which measure the systemic immune-inflammatory state, may affect the influence of VFA and SFA on the postoperative prognosis. Most importantly, cox regression analysis adjusted for confounding factors revealed that high SFA may exert its influence by promoting systemic inflammation and potentially compromising the protective effect of VFA, suggesting that its potential in aiding clinicians to devise more comprehensive intervention plans and follow-up strategies.

Despite previous studies suggesting a correlation between obesity and tumor prognosis (Barbi et al. 2021) under certain circumstances, our study found no significant differences in OS and PFS among different BMI groups. This result may be attributed to BMI’s limitation in distinguishing between muscle and fat, leading to imprecise assessments of body composition (Karra et al. 2022). Additionally, overweight benefits in elderly cancer patients are contingent on sufficient muscle mass (Gonzalez et al. 2014). To better evaluate the impact of obesity in lung cancer, the measurement of visceral fat based on CT imaging is more appropriate and informative for epidemiological and clinical research. Lee et al. (2022a) found that obesity and VFA are both protective factors for the prognosis of lung cancer patients. Consistent with this finding, our study identified that the prognosis of patients in high-VFA group was better than low-VFA group. Accordingly, appropriately enhancing preoperative visceral fat and providing dietary advice may improve lung cancer patients’ postoperative outcomes. In contrast, our study found no significant differences in OS and PFS based on SFA stratification. This result may be attributed to the metabolic stability and lipolysis resistance of subcutaneous fat compared to visceral fat (Kim et al. 2021), and advanced NSCLC with obvious malignant status was not included in our study. Furthermore, the large difference in the male-to-female ratio among NSCLC subjects, coupled with variations in subcutaneous fat distribution between genders, may have influenced the results, requiring subsequent gender-stratified studies with large samples.

SII and AISI have emerged as promising prognostic indicators in cancer patients (Wang et al. 2019a, 2023). Our study found that SII and AISI were risk factors for postoperative prognosis in NSCLC patients, indicating that SII and AISI hold promise as clinical biomarkers for predicting postoperative survival outcomes in NSCLC patients to a certain extent. This finding may be explained by the idea that SII and AISI may be indicators of the balance between pro- and anti-tumor immune status as well as the body’s reaction to systemic inflammation (Grivennikov et al. 2010; Liu et al. 2019). Consequently, preoperatively, considering the levels of SII and AISI, the use of anti-inflammatory or immunomodulatory drugs could be contemplated to regulate inflammation and immune responses. This intervention might aid in reducing the risk of tumor recurrence post lung cancer surgery and enhance patient survival. Their advantage of repeatability supports a more precise dynamic assessment of patient prognosis.

To investigate the independent impact of body composition and inflammatory markers on postoperative prognosis, Cox regression analyzes were conducted with adjustments for confounding factors. Kaplan–Meier analysis revealed that NSCLC patients without sarcopenia had significantly longer OS and PFS compared to those with sarcopenia. This underscores the importance of sarcopenia as a pivotal predictor for adverse postoperative prognosis in NSCLC patients, consistent with previous findings by Hervochon et al. (2017). Therefore, to exclude the effect of sarcopenia on other covariates, we base-adjusted for sarcopenia along with age, sex, and TMN stage. After accounting for confounding factors, different VFA groups, AISI, and SII remained significantly associated with postoperative prognosis, highlighting the potential of VFA, AISI, and SII as meaningful prognostic factors for OS and PFS. Notably, after additional adjustment for VFA, SFA emerged as a risk factor for OS in patients, suggesting an intricate interplay between VFA, SFA, and their derived adipose factors in tumor-related processes, thereby influencing patient survival prognosis. In our study, after additional correction of SFA (Cox regression model V), different groups of VFA were still associated with postoperative prognosis of patients, but the risk effect of low VFA (HR = 2.538) was increased compared with other Cox regression models, suggesting that the mechanism of obesity affecting postoperative prognosis of patients is not single. This may be due to the fact that obesity exerts systemic effects on various tissues, leading to increased levels of non-esterified fatty acids (NEFAs), insulin, leptin, and inflammatory factors, as well as decreased adiponectin levels (Font-Burgada et al. 2016; Spoto et al. 2023). Dysregulation of these factors collectively influences tumor development and prognosis.

To delve deeper into the interplay between VFA and SFA and their impact on postoperative survival in NSCLC patients, we conducted a stratified survival analysis based on VFA and SFA. Our findings revealed significant differences in the distribution of SII and AISI and survival prognosis among the four subgroups based on VFA and SFA. This implies that the systemic immune-inflammatory status linked to SII and AISI may modulate the effects of obesity and/or adipose tissue on survival prognosis (Rathmell 2021). Furthermore, our study demonstrated that patients in the high VFA & low SFA group exhibited the lowest levels of inflammatory markers and the most favorable postoperative prognosis. This suggests that VFA and SFA may exert distinct effects on inflammatory markers, with high VFA potentially inhibiting systemic inflammation while high SFA may promote inflammation, thereby influencing the mutual predictive significance on survival prognosis. Therefore, effectively controlling subcutaneous fat content preoperatively could potentially reduce postoperative complications, enhance surgical success rates, and improve overall patient recovery. In addition, under different VFA (high/low) stratification, we still concluded that low SFA is a protective factor for patients’ OS, which also supports the above view. According to Lee et al.’s retrospective cohort research in patients with metastatic melanoma, SII influenced the impact of visceral fat on prognosis during immune therapy and displayed a negative correlation with visceral fat index (Lee et al. 2022b), which is consistent with our findings. The analysis suggests that obesity-induced inflammation in tumor progression can induce T cell aging and activate inhibitory pathways to counter chronic inflammatory states (Wang et al. 2019b). Moreover, studies have indicated that subcutaneous adipose tissue in obese patients exhibits significantly higher expression of pro-inflammatory genes compared to visceral fat tissue, with a higher expression intensity of IL6 and IL8 in visceral fat (Spoto et al. 2014). And expression of inflammatory genes in subcutaneous adipose tissue is positively correlated with the corresponding gene products in the circulation. Additionally, pro-inflammatory factors secreted by subcutaneous adipocytes can recruit immune cells expressing corresponding receptors, leading to the secretion of additional pro-inflammatory mediators and autoimmune antibodies, thereby promoting and sustaining local and systemic inflammatory microenvironments (Frasca et al. 2018). These fundamental research findings support our result. This discovery may contribute to identifying high-risk NSCLC patients with poor postoperative prognosis from the perspective of fat distribution and inflammatory status, representing a significant strength of our study. In conclusion, providing individualized nutritional support and basic treatment preoperatively for lung cancer patients to optimize fat distribution and reduce inflammatory states may contribute to improving their postoperative prognosis.

Our study had some limitations. Firstly, this study is retrospective, and thus, only CT images at the L3 vertebral level were extracted for quantitative analysis of body composition. It lacks assessment of muscle function, such as strength or endurance, and the cutoff value for SMI still needs further validation in populations of different races and body types. Secondly, there may be unmeasured or residual confounding factors. Thirdly, despite being a multicenter study, the sample size in our cohort was relatively small, which may introduce selection bias and limit the generalizability of our findings. Future research should focus on elucidating the causal connection between body composition and systemic inflammation in NSCLC patients by prospective large-scale study, aiming to unravel the underlying biological mechanisms that drive their associations with clinical outcomes. These efforts will provide valuable insights into the therapeutic strategies and interventions that could improve the prognosis of NSCLC patients.

## Conclusion

In summary, VFA, SFA, SII and AISI could be used as prognostic markers in NSCLC patients after surgery. Notably, the systemic immune-inflammatory state identified by SII and AISI may influence how VFA and SFA affect postoperative prognosis, with high SFA potentially promoting systemic inflammation and undermining the protective benefits of VFA. These findings may help to identify NSCLC patients with poor prognosis from the perspective of fat distribution and inflammatory status, so as to provide them with targeted nutritional support and interventions.

## Data Availability

The data presented in this study are available on request from the corresponding author.
